# Prevalence, Intensity, and Associated Factors of *Schistosoma mansoni* among School Children in Northwest Ethiopia

**DOI:** 10.1155/2020/8820222

**Published:** 2020-11-12

**Authors:** Ayalew Jejaw Zeleke, Ayenew Addisu, Yalewayker Tegegne

**Affiliations:** Department of Medical Parasitology, School of Biomedical and Laboratory Sciences, College of Medicine and Health Sciences, University of Gondar, Gondar, Ethiopia

## Abstract

**Background:**

Schistosomiasis is one of the Neglected Tropical Diseases in Ethiopia, and its burden may show variations from time to time across different regions. Thus, this study was aimed at determining the prevalence, intensity, and associated risk factors of *Schistosoma mansoni* (*S. mansoni*) among schoolchildren in Northwest Ethiopia.

**Methods:**

A school-based cross-sectional study was conducted. A multistage sampling technique was used to select the study participants. Stool specimens were collected and examined using two-slide Kato-Katz method. Data were analyzed using SPSS version 20 software. Multivariate logistic regression analysis was used to identify risk factors. *p* values less than 0.05 were taken as statistically significant.

**Result:**

A total of 786 schoolchildren were participated in this study. The prevalence of *S. mansoni* was 33.5%. The mean egg count of the parasite among the infected study participants was 523.665 eggs per gram (epg) of stool. Thirty-seven, 42, and 21 percent of the study participant's infection were due to light, moderate, and heavy infection intensities, respectively. Age of 8-11 years old (AOR = 1,687, 95%CI = 1.163, 2.892), 5^th^-8^th^ grade level (AOR = 2.280, 95%CI = 1.348, 3.856), residing in Chuahit District (AOR = 95.559, 95%CI = 12.945, 705.419), and using untreated water for domestic supply (AOR = 1.724, 95%CI = 1.457, 2.148) were found to be risk factors for *S. mansoni* infection.

**Conclusion:**

High prevalence of *S. mansoni* and relatively higher proportion of moderate intensity of infection in this study imply that schistosomiasis is still one of the major public health problems in Northwest Ethiopia. It is also highlighted that study sites, provision of water supply, age, and grade level of the schoolchildren were identified as a risk factors for the disease.

## 1. Background

Intestinal schistosomiasis is one of the Neglected Tropical Diseases (NTD). Globally, an estimated 732 million people are at risk of this disease. It is endemic in 77 countries, and around 230 million people are infected worldwide. Recent disease burden assessment indicates that up to 70 million disability-adjusted life years (DALYs) are lost annually due to this disease in the globe [1]. It is one of the major causes of morbidity and mortality in Africa, South America, the Caribbean, the Middle East, and Asia [2].

People living in developing countries are at risk of intestinal schistosomiasis, since they live in conditions that exacerbate transmission. Endemic areas for intestinal schistosomiasis have no access to proper health care and effective prevention measures. They are often characterized by low socioeconomic conditions, poor sanitary facilities, and offensive practice of the community such as urination and defecation in canal water. Finally, people will have an exposure to this contaminated water by bathing, swimming, washing food utensils and clothes, walking on bare-foot during irrigation, or fishing [3].

School- age children are at risk of schistosomiasis for multiple reasons. For instance, children have a habit of frequent swimming, and this will make them at high risk, because of their prolonged exposure for infection. Besides, adults who travelled to endemic areas are susceptible to this parasitic infection [4–6].

School- or community-based mass drug administration (MDA) using praziquantel is the major control strategy for schistosomiasis. Similar to other endemic countries, the MDA strategy in Ethiopia mainly focuses on school-aged children via yearly school-based treatment. This campaign started in 2015 and is currently targeting 6.4 million children in endemic areas countrywide. The Ethiopian Ministry of Health (MoH) has planned to achieve the elimination of schistosomiasis-related morbidity by 2020 and to break the transmission by 2025 [7]. Short-term targets of the elimination program are to cover at least 75% of school-aged children with MDA, to extend MDA to all adolescents and adults in high-endemic districts, and to decrease infection rates by 65-90% compared to baseline estimates [8, 9]. Therefore, a continuous evaluation of the burden of the disease linked with the major prevention and control measures is essential.

Nowadays, several prevalence studies on intestinal schistosomiasis and other parasitic infections have been conducted in Ethiopia [5, 10–12]. However, its prevalence among schoolchildren in these study areas has not well addressed. Therefore, the aim of this study was to determine the prevalence, infection intensity, and associated risk factors of *S. mansoni* infection among schoolchildren from four districts around Gondar Town, Northwest Ethiopia. The finding of this study will possibly strengthen the evidences that are already available hitherto for scaling up and designing of an effective communication strategy in fighting of schistosomiasis in the study districts.

## 2. Methods

### 2.1. Study Design, Area, and Population

A school-based cross-sectional study was conducted among school-aged children (SAC) of Maksegnit, Debark, Sanja, and Chuahit districts from January 21 to February 21 2018. These are among the districts in Amhara region, Northwest Ethiopia. The topography of the districts shows mountains and plain land with rivers, streams, and springs which are often used as source of water for home and other uses by the communities. As a result of these, children living in such districts are at risk of developing water-borne diseases during swimming, washing, playing, and crossing the water.

### 2.2. Sample Size Determination and Sampling Technique

**Figure 1:**
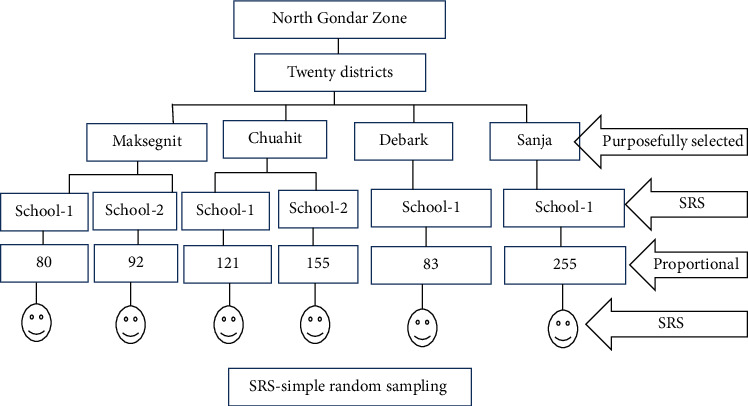
Schematic presentation of sampling technique.

The sample size was calculated by using a single proportion formula by considering the following assumption: using a prevalence of 50%, 95% confidence level, 5% margin of error, design effect of 2, and 5% for anticipated nonresponse rate. Accordingly, the minimum sample size (*n*) was found to be 786 school children. A multistage sampling technique was used for study participant selection. Considering logistics and other resources, four districts from Northwest Ethiopia were purposively selected. After that, the number of schools involved in each of the districts was selected randomly. Proportional sample allocation was deployed. Finally, using class roster list as a sampling frame, systematic random sampling technique was used to select the study participants from each school (Figure 1).

## 3. Data Collection and Processing

### 3.1. Questioner Survey

Trained medical laboratory professionals were used for the data collection. A pretested questionnaire which is written in Amharic language was used to collect data. Information regarding sociodemographic characteristics of study participants, grade, source of drinking water, house hold latrine availability, habit of wearing shoe, history of taking bath in the river, and history of having antihelminths drug was collected by interviewing the SAC and their parents.

### 3.2. Sample Collection and Laboratory Procedures

School age children, without having any kind of chronic infection, who had no any history of taking antihelminths drug in the past four weeks and volunteer to give a stool sample, were included in the study. A clean, dry, and leak-proof container was used to collect a stool specimen of about 5 g from each of the SAC. Then, the stool samples were transported to the University of Gondar Research Laboratory and was processed by two-slide Kato-Katz technique [13]. The intensity of *S. mansoni* infection was calculated to determine the worm burden based on the intensity classes set by WHO as light (1–99 epg), moderate (100–399 epg), and heavy (epg > 400) infection [8].

### 3.3. Data Management and Analysis

Data were analyzed by SPSS version 20 software. Sex, age, study site, and grade of SAC were presented as frequencies and percentages. Binary logistic regression analysis was used to determine the risk factors associated with the prevalence of *S. mansoni* infection. Chi-square test was used to determine some associations with the prevalence of *S. mansoni.p* values less than 0.05 were considered as statistically significant.

## 4. Result

### 4.1. Sociodemographic and Other Characteristics

A total of 786 schoolchildren were included in the present study. The mean age of the study subjects was 10.64 ± 1.8years with a minimum and maximum age of 7 and 16 years, respectively. In terms of sex, the number of females was 452 (52.4%). The study participants were from the four different districts. Accordingly, 21.9%, 10.6%, 32.4%, and 35.1% were from Maksegnit, Debark, Sanja, and Chuahit districts, respectively. Most of the study participants did not have treated source of water for drinking (38.5%) and latrine at home (33.3%). Moreover, the majority of SAC had the habit of taking baths in rivers. The other sociodemographic and behavioral characteristics of the study participants are summarized in Table 1.

### 4.2. Prevalence of *S. mansoni* Infection across Districts

As it is shown below (Table 2), 263 out of 786 (33.5%) were found to be infected by *S. mansoni*. The prevalence of the parasite showed a statistically significant differences (*p* < 0.001) across the four districts. The highest prevalence was observed in Chuahit (51.8%) followed by Sanja (38%). The least prevalence was seen in Debark District.

### 4.3. Intensity of *S. mansoni* Infection

The mean egg count of the parasite among the infected participants was found to be 523.665 epg. Thirty-seven, 42, and 21 percent of the infections were due to light, moderate, and heavy intensity of infections, respectively (Table 3).

### 4.4. Risk Factors Associated with *S. mansoni* Infection

The multivariate logistic regression analysis showed that age, grade level, study districts, and source of drinking water were found to be risk factors for *S. mansoni* infection. Children who had within 8-11 and greater than 11 years of age were 1.5 (AOR = 1,687, 95%CI = 1.163, 2.892), and 2 (AOR = 2.147, 95%CI = 1.485, 9.511) times more likely to be infected by the parasite than those whose age was 7 years and below, respectively. Study participants in which their grade is from 5^th^-8^th^ were 2 times (AOR = 2.280, 95%CI = 1.348, 3.856) more risky than those who were within 1-4 grade levels. Moreover, children who were residing in Chuahit and using untreated water for drinking were 95 (AOR = 95.559, 95%CI = 12.945, 705.419) and 1.5 (AOR = 1.724, 95%CI = 1.457, 2.148) times more risky for the parasite infection than their counterparts, respectively (Table 4).

## 5. Discussion

The burden of intestinal schistosomiasis has been determined in different parts of Ethiopia [4–6]. However, there are still many areas whose prevalence, intensities of infection, and associated risk factors have not yet addressed. In the current study, the overall prevalence of *S. mansoni* infection is found to be 33.5%. This is in line with the previous study carried out in one of the current study districts (33.7%) [14]. On the other hand, the prevalence rate of *S. mansoni* infection in the present study was higher than previous studies carried out from other parts of Ethiopia including Amibera (0.8%) [15], Gondar (4%) [16], Gorgora (20.6%) [17], Jimma (27.6%) [18], and Gelgel Gibe area [6]. It is also higher than studies conducted from elsewhere outside Ethiopia such as Ghana (19.8%) [19], Yemen (9.3%) [20], and Sudan (2.95%) [21]. The existence of high prevalence of *S. mansoni* in the study area could suggest extra and aggressive intervention measures are needed. However, it is lower than studies conducted in different parts of Ethiopia such as Wolita (81.3%) [22], Sanja (89.9%) [4], Mizan Teferi (44.8%) [23], Tumuga (73.9%) [24], and Wollega (67.9%) [25] and from countries other than Ethiopia, namely, Tanzania (84.01%) [26] and Kenya (76.8%) [27]. The observed differences in the prevalence rates among studies could be due to difference in the distribution of snail vector (*Biomphalaria pfeifferi*) for the transmission of the disease, climatic condition, study period, and frequency of contact with water by the people.

The proportions of light, moderate, and heavy intensities of *S. mansoni* infection in this study were 37, 42, and 21%, respectively. This is in line with the previous reports from Tanzania [26] and from Yemen [26]. The existence of a significant number of moderate and heavy infections in this study may indicate that there is a frequent reinfection of schoolchildren by the parasite and this might be due to prolonged exposure to the water source. It may also imply that the presence of a considerable number of morbidity and other bad health consequences among the schoolchildren in particular and in the whole community in general.

The present study showed that a higher proportion of males were infected by *S. mansoni* than females. However, the difference was not statistically significant. This finding is in agreement with studies conducted elsewhere in the world [28, 29]. It may indicate that both males and females have similar exposure to the infective stage of the parasite. On the other hand, the prevalence of *S. mansoni* and age showed statistically significant association. Thus, children in the age group 11 years and above had the highest infection rate compared to the rest of the age groups and was just 2 times more likely to be positive for *S. mansoni* than those whose age were 7 years and below. This is in line with a previous report, which indicated that children in the age group of 10–14 years had relatively higher infection rate than children below 9 years of age [30]. This may suggest that as age of the children increases, the rate of exposure for the parasite will be greater than before this might be due to as children gets older their chance of playing outside the home like frequency of swimming in the river increase. Grade (educational) level of the schoolchildren was also an independent predictor for *S. mansoni* infection. As a result, schoolchildren, whose grade level of four and below, were less likely for the parasite infection than those who were within 5-8 grade levels. Instead, this might be linked with the age of the child in which children who were within 5^th^ to 8^th^ grade levels were older than others, and the current study had already identified that age is one of the risk factors for the disease as stated above.

The finding of the present study also demonstrated that the prevalence of *S. mansoni* showed a statistically significant differences (*p* < 0.001) across the four districts. Accordingly, the highest prevalence was observed in Chuahit (51.8%), and residing in this study site was just 95 times more likely to be infected by the parasite than living in Debark District (1.2%). Sanja was the second highest district on the parasite prevalence (38%). Higher prevalence of schistosomiasis in Chuahit and Sanja districts could be due to the existence of rivers in which the communities have been using for washing of clothes, taking of baths, and fetching of water for domestic purpose. These rivers may serve as a potential source of infection for *S. mansoni.* Moreover, the weather condition in these two districts is relatively warmer and more humid than the others. Hence, this may favor the existence and reproduction rate of the disease transmitter snail vector. The other possible reasons behind the discrepancies in the infection rate of the disease among districts might be related with the differences in children's behavior to contact with cercaria-infested water and level of awareness about the prevention and control of *S. mansoni* infection [7].

The major limitation of this study is that infection intensity of *S. mansoni* was determined by examination of single stool specimen, and this might have affected the accuracy of *S. mansoni* egg detection and count.

## 6. Conclusion

Both the prevalence and intensity of *S. mansoni* infection was alarming. This indicates that *S. mansoni* is still among the major public health problems among schoolchildren in the study areas. The prevalence was disproportionately higher in Chuahit and Sanja than the other two districts. It is also highlighted that provision of water supply, age, and grade level of the schoolchildren were identified as a risk factors for the disease. Thus, we recommend immediate and integrated schistosomiasis prevention and control measures in Northwest Ethiopia.

## Figures and Tables

**Table 1:** Sociodemographic, behavioral characteristics of schoolchildren in Northwest Ethiopia, 2018.

Variables, *n* = 786	Frequency, *n* (%)	Variables, *n* = 786	Frequency, *n* (%)
Sex		Household latrine availability	
Male	374 (47.6)	Yes	524 (66.7)
Female	412 (52.4)	No	262 (33.3)
Age		Habit of wearing shoe	
≤7	17 (2.2)	Wearing slipper	699 (88.9)
8-11	537 (68.3)	Wearing closed shoe	79 (10.1)
>11	232 (29.5)	Never wear	8 (1)
Study sites		Habit of taking baths in rivers	
Maksegnit	172 (21.9)	Never	234 (29.8)
Debark	83 (10.6)	Yes	552 (70.2)
Sanja	255 (32.4)	History of taking antihelminthiasis	
Chuahit	276 (35.1)	Yes	655 (83.3)
Grade		No	131 (16.7)
1-4	207 (26.3)	Source of water	
5-8	579 (73.7)	Not treated	303 (38.5)
		Treated	483 (61.5)

**Table 2:** Prevalence of *S. mansoni* in schoolchildren among study sites in Northwest Ethiopia, 2018.

Study district	*S. mansoni* infection status			Chi-square (*p* value)
	Positive, *n* (%)	Negative, *n* (%)	Total	
Maksegnit	22 (12.8)	150 (87.2)	172 (100)	115.94 (<.0001)
Debark	1 (1.2)	82 (98.8)	83 (100)	
Sanja	97 (38)	158 (62)	255 (100)	
Chuahit	143 (51.8)	133 (48.2)	276 (100)	
Total	263 (33.5)	523 (66.5)	786 (100)	

**Table 3:** *S. mansoni* infection intensity in schoolchildren in Northwest Ethiopia, 2018.

Parasite species	Infection intensity			
	Light *n* (%)	Moderate *n* (%)	Heavy *n* (%)	Mean egg count (epg)
*S. mansoni* (*n* = 263)	97 (36.9)	110 (41.8)	56 (21.3)	523.665

epg: eggs per gram.

**Table 4:** Risk factors of *S. mansoni* infection among the schoolchildren (*n* = 786) in Northwest Ethiopia, 2018.

Variables	*S. mansoni* infection status		COR (95% CI)	AOR (95% CI)
	Positive, *n* (%)	Negative, *n* (%)		
Sex				
Male	141 (37.7)	233 (62.3)	1.44 (1.068, 1.937)	1.094 (0.771, 1.553)
Female	122 (29.6)	290 (70.4)	1	1
Age				
≤7	3 (17.6)	14 (82.4)	1	1
8-11	160 (29.8)	377 (70.2)	1.981 (1.561, 6.986)	1.687 (1.163, 2.892)
>11	100 (43.1)	132 (56.9)	3.535 (1.989, 12.636)	2.147 (1.485, 9.511)
Study sites				
Maksegnit	22 (12.8)	150 (87.2)	12.027 (1.592, 90.843)	15.408 (1.992, 119.186)
Debark	1 (1.2)	82 (98.8)	1	1
Sanja	97 (38)	158 (62)	50.342 (6.895, 367.548)	41.941 (5.354, 328.559)
Chuahit	143 (51.8)	133 (48.2)	88.165 (12.101, 642.379)	95.559 (12.945, 705.419)
Grade				
1-4	49 (23.7)	158 (76.3)	1	1
5-8	214 (37)	365 (63)	1.891 (1.316, 2.716)	2.280 (1.348, 3.856)
Drinking water				
Not treated	130 (42.9)	173 (57.1)	1.977 (1.461, 2.676)	1.724 (1.457, 2.148)
Treated	133 (27.5)	350 (72.5)	1	1
Latrine				
Yes	165 (31.5)	359 (68.5)	1	1
No	98 (37.4)	164 (62.6)	1.300 (0.953, 1.774)	0.884 (0.620, 1.261)
Wearing shoe				
Slipper	234 (33.5)	465 (66.5)	1.026 (0.625, 1.683)	1.168 (0.656, 2.080)
Closed shoe	26 (32.9)	53 (67.1)	1	1
Never wear	3 (37.5)	5 (62.5)	1.223 (0.271, 5.516)	1.987 (0.285, 13.856)
Baths in river				
Never	60 (25.6)	174 (74.4)	1	1
Some times	117 (36.6)	203 (63.4)	1.671 (1.153, 2.423)	1.253 (0.770, 2.037)
Always	86 (37.1)	146 (62.9)	1.708 (1.149, 2.539)	1.043 (0.576, 1.890)
Previous treatments				
Yes	217 (33.1)	438 (66.9)	1	1
No	46 (35.1)	85 (64.9)	1.092 (0.737, 1.620)	0.717 (0.448, 1.147)

## Data Availability

All data generated or analyzed during this study are included in this published article.

## References

[1] Hotez P. J., Fenwick A. (2009). Schistosomiasis in Africa: an emerging tragedy in our new global health decade. *PLOS Neglected Tropical Diseases*.

[2] Soares Magalhães R. J., Biritwum N.-K., Gyapong J. O. (2011). Mapping helminth co-infection and co-intensity: geostatistical prediction in Ghana. *PLoS Neglected Tropical Diseases*.

[3] Sibomana L. (2010). *Association of schistosomiasis prevalence with socio-demographic status measures in Sub-Saharan Africa*.

[4] Worku L., Damte D., Endris M., Tesfa H., Aemero M. (2014). Schistosoma mansoniInfection and associated determinant factors among school children in Sanja Town, Northwest Ethiopia. *Journal of Parasitology Research*.

[5] Tsegaye S. (2005). *Determining the prevalence of intestinal parasites and associated risk factors in Yebu Elementary School Students, Jimma Zone, South West Ethiopia*.

[6] Yami A., Mamo Y., Kebede S. (2011). Prevalence and predictors of intestinal helminthiasis among school children in Jimma zone; a cross-sectional study. *Ethiopian Journal of Health Sciences*.

[7] Organization WH (2013). *Schistosomiasis: progress report 2001-2011, strategic plan 2012-2020*.

[8] Organization WH (2002). *Prevention and control of schistosomiasis and soil-transmitted helminthiasis*.

[9] Stothard J. R., Sousa-Figueiredo J. C., Khamis I. S., Garba A., Rollinson D. (2009). Urinary schistosomiasis-associated morbidity in schoolchildren detected with urine albumin-to-creatinine ratio (UACR) reagent strips. *Journal of Pediatric Urology*.

[10] Jemaneh L. (2001). Soil-transmitted helminth infections and Schistosomiasis mansoni in school children from Chilga District, northwest Ethiopia. *Ethiopian Journal of Health Sciences*.

[11] Leykun J. (1997). Intestinal helminthic infection with special reference to S. mansoni in school children in Adarkay District, North West Ethiopia. *Ethiopian Journal of Health Development*.

[12] Mohammed A. (2003). *Prevalence of intestinal parasites Kito Elementary School children in Jimma Town, South West Ethiopia*.

[13] Montresor A., Crompton D. W., Hall A., Bundy D., Savioli L., Organization WH (1998). *Guidelines for the Evaluation of Soil-Transmitted Helminthiasis and Schistosomiasis at Community Level: A Guide for Managers of Control Programmes*.

[14] Mathewos B., Alemu A., Woldeyohannes D. (2014). Current status of soil transmitted helminths and Schistosoma mansoni infection among children in two primary schools in North Gondar, Northwest Ethiopia: a cross sectional study. *BMC Research Notes*.

[15] Awoke W., Bedimo M., Tarekegn M. (2013). Prevalence of schistosomiasis and associated factors among students attending at elementary schools in Amibera District, Ethiopia. *Open Journal of Preventive Medicine*.

[16] Gelaw A., Anagaw B., Nigussie B. (2013). Prevalence of intestinal parasitic infections and risk factors among schoolchildren at the University of Gondar Community School, Northwest Ethiopia: a cross-sectional study. *BMC Public Health*.

[17] Essa T., Birhane Y., Endris M., Moges A., Moges F. (2013). Current status of Schistosoma mansoni infections and associated risk factors among students in Gorgora town, Northwest Ethiopia. *ISRN Infectious Diseases*.

[18] Bajiro M., Tesfaye S. (2017). Schistosoma mansoni infection prevalence and associated determinant factors among school children in Mana District, Jimma Zone, Oromia Region, South West Ethiopia. *Journal of Bacteriology & Parasitology*.

[19] Anto F., Asoala V., Adjuik M. (2013). Water contact activities and prevalence of schistosomiasis infection among school-age children in communities along an irrigation scheme in rural Northern Ghana. *Journal of Bacteriology & Parasitology*.

[20] Sady H., Al-Mekhlafi H. M., Mahdy M. A., Lim Y. A., Mahmud R., Surin J. (2013). Prevalence and associated factors of schistosomiasis among children in Yemen: implications for an effective control programme. *PLoS Neglected Tropical Diseases*.

[21] Hajissa K., Muhajir A. E. M. A., Eshag H. A. (2018). Prevalence of schistosomiasis and associated risk factors among school children in Um-Asher Area, Khartoum, Sudan. *BMC Research Notes*.

[22] Alemayehu B. T. Z. (2015). Schistosoma mansoni infection prevalence and associated risk factors among schoolchildren in Demba Girara, Damot Woide District of Wolaita Zone, Southern Ethiopia. *Asian Pacific Journal of Tropical Medicine*.

[23] Jejaw A., Zemene E., Alemu Y., Mengistie Z. (2015). High prevalence of Schistosoma mansoni and other intestinal parasites among elementary school children in Southwest Ethiopia: a cross-sectional study. *BMC Public Health*.

[24] Dejenie T., Asmelash T., Abdelkadir M. (2010). Efficacy of praziquantel in treating Schistosoma mansoni infected school children in Tumuga and Waja, North Ethiopia. *Momona Ethiopian Journal of Science*.

[25] Haile S., Golassa L., Mekonnen Z. (2012). Prevalence of Schistosoma mansoni and effectiveness of Praziquantel in school children in Finchaa valley, Ethiopia. *Journal of Parasitology and Vector Biology*.

[26] Munisi D. Z., Buza J., Mpolya E. A., Kinung’hi S. M. (2016). Intestinal schistosomiasis among primary schoolchildren in two on-shore communities in Rorya district, northwestern Tanzania: prevalence, intensity of infection and associated risk factors. *Journal of Parasitology Research*.

[27] Nagi S., Chadeka E. A., Sunahara T. (2014). Risk factors and spatial distribution of Schistosoma mansoni infection among primary school children in Mbita District, Western Kenya. *PLoS Neglected Tropical Diseases*.

[28] Garba A., Barkiré N., Djibo A. (2010). Schistosomiasis in infants and preschool-aged children: infection in a single Schistosoma haematobium and a mixed S. haematobium–S. mansoni foci of Niger. *Acta Tropica*.

[29] Ahmed A. M., Abbas H., Mansour F. A., Gasim G. I., Adam I. (2012). Schistosoma haematobium infections among schoolchildren in central Sudan one year after treatment with praziquantel. *Parasites & Vectors*.

[30] Haftu D., Deyessa N., Agedew E. (2014). Prevalence and determinant factors of intestinal parasites among school children in Arba Minch town, Southern Ethiopia. *American Journal of Health Research*.

